# Peripheral Leukocyte TET Protein Expression Is Associated with Plasma Coagulation Parameters in Patients with Heart Failure with Reduced Ejection Fraction: A Two-Center Cross-Sectional Study

**DOI:** 10.3390/jcm15155942

**Published:** 2026-07-30

**Authors:** Anna Wołowiec, Łukasz Wołowiec, Grzegorz Grześk, Jacek Budzyński, Joanna Banach, Joanna Osiak-Gwiazdowska, Albert Jaśniak, Paulina Jakubowska, Mariusz Kozakiewicz

**Affiliations:** 1 Department of Geriatrics, Faculty of Health Sciences, Ludwik Rydygier Collegium Medicum in Bydgoszcz, Nicolaus Copernicus University in Toruń, 85-094 Bydgoszcz, Poland; 2 Department of Cardiology and Clinical Pharmacology, Faculty of Health Sciences, Ludwik Rydygier Collegium Medicum in Bydgoszcz, Nicolaus Copernicus University in Toruń, 85-168 Bydgoszcz, Poland; lukasz.wolowiec@cm.umk.pl (Ł.W.); g.grzesk@cm.umk.pl (G.G.); jbanach@cm.umk.pl (J.B.); 503677@doktorant.umk.pl (J.O.-G.); albert.jasniak@cm.umk.pl (A.J.); 3 Department of Vascular and Internal Diseases, Faculty of Health Sciences, Ludwik Rydygier Collegium Medicum in Bydgoszcz, Nicolaus Copernicus University in Toruń, 85-094 Bydgoszcz, Poland; jb112233@cm.umk.pl; 4 Department of Cardiology and Cardiac Surgery, 10th Military Clinical Hospital, 85-681 Bydgoszcz, Poland; paujaku@wp.pl

**Keywords:** heart failure with reduced ejection fraction, TET proteins, direct oral anticoagulants, activated partial thromboplastin time, immunothrombosis, flow cytometry, international normalized ratio, blood coagulation

## Abstract

**Background/Objectives**: Heart failure with reduced ejection fraction (HFrEF) involves coexisting inflammatory and hemostatic dysregulation contributing to thromboembolic and bleeding risk. Ten-eleven translocation (TET1-3) proteins regulate immune-cell phenotype, but their relationship to plasma coagulation parameters in HFrEF is poorly defined. We aimed to determine whether TET1-3 protein expression in peripheral blood leukocyte subpopulations is associated with routinely available plasma coagulation parameters in HFrEF, and whether associations differ by direct oral anticoagulant (DOAC) exposure and clinical phase. **Methods**: In this cross-sectional, observational, two-center study, 107 patients with HFrEF were enrolled (39 receiving a DOAC, non-randomized; 68 not receiving a DOAC, of whom seven received a vitamin K antagonist and 61 received no oral anticoagulant). TET1-3 protein expression was assessed by flow cytometry in T lymphocytes, B lymphocytes, natural killer (NK) cells, monocytes, and granulocytes, expressed as the ratio of test-to-negative-control geometric mean fluorescence intensity. Associations with activated partial thromboplastin time (aPTT) and international normalized ratio (INR) were analyzed using nonparametric tests and Spearman correlations; DOAC samples were drawn at steady-state trough. **Results**: DOAC-treated patients constituted a distinct clinical subgroup (older, predominantly AF/AFL) and showed longer aPTT and higher TET expression in selected immune-cell subsets; these between-group differences cannot be attributed to DOAC therapy. Patients with aPTT at or above the median showed higher TET expression in T cells, B cells, NK cells, and selected leukocyte indices, whereas INR-based stratification did not. In continuous analyses, TET indices showed multiple moderate positive Spearman correlations (R = 0.25–0.49) with both aPTT and INR—with INR showing the strongest single association—and no associations with composite inflammatory indices (NLR, SII, SIRI); all comparisons were exploratory and uncorrected for multiplicity. Associations appeared stronger in stable than in worsening HFrEF (exploratory). **Conclusions**: Peripheral leukocyte TET protein expression was associated with plasma coagulation parameters in HFrEF; the aPTT-specific pattern was confined to the categorical (median-split) analysis, whereas continuous analyses showed associations with both aPTT and INR. Because of the cross-sectional design, moderate sample size, and potential confounding by indication, these findings should be considered hypothesis-generating. TET-related immune-cell phenotyping may capture a non-redundant immune–coagulation signal in HFrEF and warrants prospective validation.

## 1. Introduction

Heart failure with reduced ejection fraction (HFrEF) remains a leading cause of morbidity and mortality, with global estimates exceeding 60 million affected individuals [1]. Contemporary models extend beyond hemodynamics and recognize HFrEF as a syndrome of persistent inflammatory activation, endothelial dysfunction, and disturbed hemostatic balance that intensify with disease progression; inflammatory burden has been shown to stratify the risk of heart failure hospitalization across the Universal Definition of Heart Failure [2]. Clinically, these disturbances translate into a dual and often competing burden of thromboembolic and bleeding risk, and existing tools capture this immune–hemostatic interface poorly. There is therefore a need for clinically interpretable biomarkers that integrate immune and hemostatic information in HFrEF.

The prothrombotic state in heart failure—activation of coagulation and platelets, endothelial dysfunction, and stasis related to reduced cardiac output—can be framed within the concept of immunothrombosis, the bidirectional coupling of inflammatory and coagulation pathways [3,4,5]. Within this framework, immune-cell populations including T and B lymphocytes, natural killer (NK) cells, and monocytes actively shape the prothrombotic phenotype [6,7], and activated lymphocytes and NK cells release increased numbers of extracellular vesicles in chronic heart failure [8]. This interface is particularly relevant where anticoagulation decisions, especially in coexisting atrial fibrillation (AF), intersect with inflammatory biology.

Direct oral anticoagulants (DOACs)—direct factor Xa and thrombin inhibitors—are clinically indicated anticoagulants in many patients with HFrEF, particularly those with AF or atrial flutter (AFL). Beyond anticoagulation, experimental studies suggest that DOACs may influence cellular signaling at the interface of inflammation and hemostasis, and DOAC exposure has been associated with anti-inflammatory, barrier-protective, and remodeling-modulating effects and with altered extracellular-vesicle protein cargo [9,10,11,12,13,14,15,16,17,18,19,20]. The clinical relevance of these pleiotropic effects remains uncertain, and these observations are hypothesis-generating rather than evidence of a direct cellular effect in patients.

Ten-eleven translocation (TET1–3) proteins regulate transcriptional programs and immune-cell plasticity and have been implicated in the differentiation and function of T cells, B cells, and NK cells [21,22,23,24,25]. TET2-related clonal hematopoiesis of indeterminate potential has been linked to heart failure progression, to incident heart failure with preserved ejection fraction, and to AF risk through inflammasome-dependent pathways [26,27,28,29,30]. In the present study, we measured TET1–3 protein expression in circulating leukocytes by flow cytometry; we did not measure DNA methylation, 5-hydroxymethylcytosine, TET enzymatic activity, or clonal hematopoiesis. TET protein expression is therefore best regarded here as a marker of immune-cell phenotype rather than as a direct epigenetic effector.

Despite extensive work on immunothrombosis, the pleiotropic effects of DOACs, and the role of the TET axis in immunity, studies linking TET protein expression in immune cells with plasma coagulation parameters in HFrEF—while accounting for DOAC exposure and clinical phase—are lacking. This gap is clinically meaningful because risk stratification in AF still relies largely on the CHA_2_DS_2_-VASc and HAS-BLED scores, which have only moderate discrimination, whereas biomarker-based approaches may improve prediction [31,32].

We aimed to determine whether TET1–3 protein expression in peripheral blood leukocyte subpopulations is associated with plasma coagulation parameters in patients with HFrEF and whether these associations differ according to DOAC exposure and clinical phase. We hypothesized that TET protein expression would be associated with routinely available plasma coagulation parameters (aPTT and INR), and we examined whether the pattern of association differed between categorical (median-split) and continuous analyses.

## 2. Materials and Methods

### 2.1. Study Design and Population

This was a cross-sectional, observational, two-center study including 107 patients with HFrEF, of whom 39 (36.4%) received DOACs according to clinical indications, whereas 68 (63.6%) were not receiving a DOAC (of whom 7 were receiving a vitamin K antagonist) (Table 1). Patients were recruited from the Department of Cardiology and Clinical Pharmacology, Ludwik Rydygier Collegium Medicum in Bydgoszcz (Biziel University Hospital), and from the Department of Cardiology and Cardiac Surgery, 10th Military Clinical Hospital in Bydgoszcz. HFrEF was diagnosed according to European Society of Cardiology (ESC) criteria, and patients received guideline-directed medical therapy [33]. DOAC therapy was clinically indicated and was not randomized; consequently, DOAC exposure is susceptible to confounding by indication, in particular by atrial fibrillation.

Inclusion criteria were age ≥ 18 years, chronic heart failure diagnosed according to ESC guidelines, New York Heart Association (NYHA) class II–IV, and left ventricular ejection fraction (LVEF) ≤ 40% assessed during the index hospitalization or within the preceding 6 months. Exclusion criteria were sepsis or shock of any etiology on admission, acute coronary syndrome, recent (<3 months) myocardial infarction or stroke, active malignancy, autoimmune disease, impaired liver function (INR without anticoagulation > 1.5, total bilirubin > 1.5 mg%, or alanine aminotransferase > 3× the upper limit of normal), systemic corticosteroid therapy, decompensated diabetes mellitus requiring intravenous insulin, chronic inflammatory bowel disease, or recent (<3 months) surgery.

### 2.2. Clinical Definitions

Clinical stability was defined as the absence of worsening heart failure signs or symptoms and no need for intensification of therapy (escalation of oral diuretics, intravenous vasoactive or diuretic treatment, an urgent heart failure visit, or unplanned hospitalization) within the 4 weeks preceding blood sampling. Patients not meeting these criteria were classified as having worsening HFrEF, or acute decompensated HFrEF if hospitalized; classification was based on the treating cardiologist’s assessment at sampling. Atrial fibrillation/flutter (AF/AFL) was defined by documented electrocardiographic or device evidence. Rehospitalization was defined as any unplanned cardiovascular admission within 12 months, and one-year mortality as all-cause death within 12 months of enrolment.

### 2.3. DOAC Exposure and Blood Sampling

Peripheral venous blood was collected during routine clinical assessment. In DOAC-treated patients, blood was collected under steady-state conditions at the trough level, i.e., within 30 min before the scheduled next dose; trough sampling was chosen to minimize pharmacodynamic distortion of coagulation tests, because DOAC concentrations are lowest at this point in the dosing cycle [34,35]. Participants had received a DOAC for at least 6 months, with uninterrupted use at a stable dose for at least the preceding 4 weeks. Blood was collected into EDTA tubes containing TransFix™ reagent (Cytomark Ltd., Buckingham, UK), which arrests cellular metabolism at collection and stabilizes surface antigens. Because DOAC exposure was clinically indicated and not randomized, it is susceptible to confounding by indication.

### 2.4. Flow-Cytometric Assessment of TET1–3 Protein Expression

The indirect two-step flow-cytometric protocol used here to quantify intracellular TET1–3 protein in peripheral blood leukocytes was the method we previously established and described in full in patients with HFrEF [36], and it was applied in the present study without modification. Because no commercially validated antibodies against TET1–3 suitable for direct flow-cytometric detection were available, an indirect two-step immunofluorescent protocol was applied: unconjugated primary antibodies followed by species-specific fluorochrome-conjugated secondary antibodies, both titrated to maximize the signal-to-noise ratio and minimize nonspecific binding [37]. Primary antibodies were rabbit polyclonal anti-TET1 (ab121587, Abcam, Cambridge, UK), goat polyclonal anti-TET2 (ab99432, Abcam, Cambridge, UK), and rabbit polyclonal anti-TET3 (ab139311, Abcam, Cambridge, UK); secondary antibodies were donkey F(ab′)_2_ anti-rabbit IgG Alexa Fluor 488 (ab181346) and rabbit F(ab′)_2_ anti-goat IgG Alexa Fluor 647 (ab169347), both Abcam (1:1000). Permeabilization used the BD Cytofix/Cytoperm™ kit (BD Biosciences, San Jose, CA, USA).

Leukocytes were enumerated (LUNA-II, Logos Biosystems, Anyang-si, Gyeonggi-do, South Korea) to standardize cell-to-antibody ratios; 100,000 cells per test were surface-stained with PE-anti-CD56/CD16, PerCP-anti-CD14, APC-H7-anti-CD19, BV421-anti-CD45, and V500-anti-CD3 (BD Biosciences, San Jose, CA, USA). After erythrocyte lysis, fixation, and permeabilization, primary anti-TET antibodies were applied, followed by the appropriate secondary antibody; control tubes received surface and secondary antibodies only (negative controls). Data were acquired on a CytoFLEX cytometer (Beckman Coulter, Brea, CA, USA) after daily calibration and analyzed in CytExpert 2.4 (Beckman Coulter, Brea, CA, USA). Doublets were excluded by FSC-A/FSC-H gating; granulocytes, monocytes, and lymphocytes were identified by side scatter and CD45, and T (CD3^+^), B (CD19^+^), and NK (CD56^+^CD16^+^) cells by surface markers.

TET protein expression was expressed as the ratio of the geometric mean fluorescence intensity (GMFI) of the test sample to that of the corresponding negative control (ratio = GMFItest/GMFIcontrol) [38], which inherently normalizes for day-to-day instrument drift and nonspecific background. Percentage-weighted indices were derived by weighting the expression ratio by the proportion of cells in each subpopulation. The indirect immunofluorescence technique has been described in detail previously [39], and this TET1–3 leukocyte staining panel was applied in our prior HFrEF study [36]. The primary antibodies were used at the titration-optimized concentration of 1 µL of stock per test (a 1:100 final dilution in Perm/Wash buffer), applied for 30 min at room temperature in the dark. Each sample was accompanied by a matched secondary-only negative control (no primary antibody) carried in parallel through the identical fixation, permeabilisation and surface-staining protocol, and background fluorescence was read within the same gated leukocyte population; isotype-control staining was not performed. Antibody specificity was therefore assessed only against these matched secondary-only controls: no orthogonal validation (Western blot, ELISA, immunofluorescence microscopy or targeted proteomics in the same samples, or TET-knockout or TET-overexpressing systems), no measurement of TET enzymatic activity, and no assessment of DNA methylation or hydroxymethylation were carried out. Because the anti-TET1–3 reagents are polyclonal antibodies that were not independently re-validated in our laboratory, between-lot variability and partial cross-reactivity with structurally related epitopes cannot be formally excluded. The reported ratios should accordingly be interpreted as relative immunoreactive signals—antibody-accessible epitope abundance under the staining conditions used—rather than as analytically validated absolute quantification of catalytic enzyme, and the assay is described here as standardized to a negative control rather than as validated. This is reflected in the Limitations.

### 2.5. Coagulation and Laboratory Parameters

Hemostatic status was assessed using two routinely available plasma coagulation parameters, the activated partial thromboplastin time (aPTT) and the international normalized ratio (INR), measured as part of routine clinical laboratory testing. Platelet count was recorded within the complete blood count; although not a coagulation-time test, it is a determinant of hemostatic capacity and is therefore reported and analyzed as a distinct cellular variable. Additional parameters included complete blood count with leukocyte differential, high-sensitivity C-reactive protein (hs-CRP), N-terminal pro-B-type natriuretic peptide (NT-proBNP), renal and hepatic indices, and derived composite inflammatory indices (neutrophil-to-lymphocyte ratio [NLR], systemic immune-inflammation index [SII], systemic inflammation response index [SIRI], and related ratios). Echocardiographic parameters included LVEF, left ventricular end–diastolic diameter (LVEDD), and tricuspid annular plane systolic excursion (TAPSE). The study did not include dedicated measures of thrombin generation, fibrinolysis, endothelial activation, or platelet activation (e.g., D-dimer, fibrinogen, thrombin–antithrombin complex, prothrombin fragment 1+2, PAI-1, or von Willebrand factor); the coagulation assessment is therefore limited to aPTT and INR.

### 2.6. Clinical Follow-Up and Outcomes

All 107 participants entered a prespecified observational follow-up for clinical outcomes. Outcome ascertainment was by structured telephone contact with the patient or, in the event of death, with a first-degree relative, supplemented by hospital records for admissions and in-hospital deaths at the recruiting departments; causes were not centrally adjudicated. The prespecified outcomes were unplanned cardiovascular rehospitalization within 12 months and all-cause mortality within 12 months of enrolment (Section 2.2). Outcome data were complete for all 107 participants. These outcomes are reported as baseline cohort characteristics (Table 1); the study was not designed or powered to relate TET indices to clinical events, and no time-to-event analysis of TET expression was performed.

### 2.7. Statistical Analysis

Analyses were performed in Statistica v.13.3 (TIBCO Software Inc., Palo Alto, CA, USA). Normality was assessed using the Kolmogorov–Smirnov test. Approximately normally distributed continuous variables are presented as mean ± standard deviation and compared with the Student *t*-test; TET parameters and other non-normally distributed continuous variables are presented as median (interquartile range) and compared with the Mann–Whitney U test; categorical variables are presented as number (percentage) and compared with the chi-square or Fisher exact test. Associations between TET parameters and plasma coagulation parameters were assessed by Spearman rank correlation, with two-sided *p* < 0.05 considered nominally significant. Spearman correlations were computed on complete case-pairs (patients with both the relevant TET parameter and the coagulation parameter available); these analyses therefore refer to the paired subset rather than to all 107 patients. Between-group comparisons of TET parameters likewise used the Mann–Whitney U test; the exact *p* values reported in Table 2 and Table 3 were regenerated accordingly.

Because multiple TET parameters and subgroup comparisons were analyzed, these analyses should be interpreted as exploratory.

Patients were additionally compared according to documented AF/AFL versus its absence, using the same tests. To identify which TET parameters were associated with the coagulation parameters, forward stepwise multiple regression was performed with aPTT, INR and platelet count as dependent variables and the TET-protein parameters as independent variables. Given the number of candidate TET predictors relative to the sample size and their intercorrelation, these regression models are regarded as exploratory and hypothesis-generating.

## 3. Results

### 3.1. Baseline Clinical Phenotype According to DOAC Exposure

Of 107 patients with HFrEF, 39 (36.4%) received a DOAC and 68 (63.6%) did not (seven of the latter received a vitamin K antagonist) (Table 1). Compared with non-anticoagulated patients, those receiving DOACs were older (70.6 ± 11.0 vs. 64.3 ± 13.2 years; *p* = 0.013), more frequently had AF (66.7% vs. 10.3%; *p* < 0.001), and were more often rehospitalized within 12 months (43.6% vs. 18.9%; *p* = 0.003). Patients receiving DOACs showed longer aPTT (36.2 ± 10.2 vs. 30.8 ± 7.4 s; *p* = 0.002), lower TAPSE (*p* = 0.048), lower platelet and absolute neutrophil counts, and higher hs-CRP (12.1 ± 19.1 vs. 6.5 ± 8.9 mg/L; *p* = 0.040). Despite higher hs-CRP, the DOAC group showed lower values of the composite inflammatory indices NLR, SII, and SIRI. The between-group difference in INR did not reach significance (*p* = 0.062).

### 3.2. TET Protein Expression According to DOAC Exposure

Patients receiving DOACs showed higher TET protein expression than non-anticoagulated patients in several leukocyte subpopulations (Table 2). Higher values were observed for T- and B-lymphocyte TET1 and TET3 and for NK-cell TET1, TET2, and TET3, both as raw GMFI ratios and as percentage-weighted indices, and for selected granulocyte, monocyte, and lymphocyte indices. These are observational between-group differences and should not be interpreted as a direct effect of DOACs on TET expression.

These findings indicate that DOAC exposure identified a clinically distinct subgroup with higher TET protein expression in selected leukocyte subpopulations.

### 3.3. TET Protein Expression According to aPTT and INR

When patients were stratified by the median aPTT (30.7 s), those with aPTT ≥ 30.7 s (n = 53) showed higher TET expression than those with aPTT < 30.7 s (n = 54) across T lymphocytes, B lymphocytes, and NK cells for all three isoforms, together with higher selected monocyte- and lymphocyte-based indices (Table 3). Patients in the higher-aPTT subgroup more often received DOACs—particularly apixaban—and more frequently had a history of AF/AFL. By contrast, stratification by the median INR did not reveal significant differences in TET expression; patients with higher INR had higher NT-proBNP, lower LVEF and TAPSE, and higher NPR and SIRI.

The aPTT-based stratification revealed a more coherent TET-related pattern than INR-based stratification.

### 3.4. Correlations Between TET Protein Expression and Plasma Coagulation Parameters

**Figure 1:**
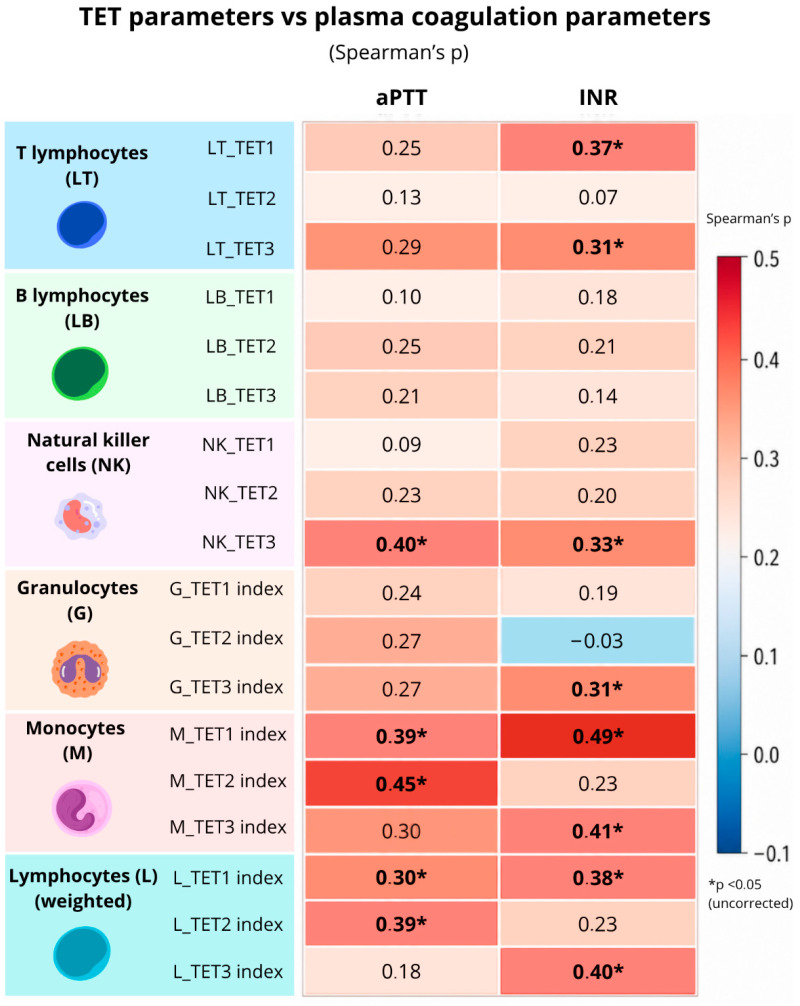
Heatmap of Spearman rank-correlation coefficients between TET parameters and the plasma coagulation parameters activated partial thromboplastin time (aPTT) and international normalized ratio (INR). Cells display Spearman’s ρ; asterisks indicate nominal statistical significance at *p* < 0.05 without correction for multiple comparisons. Correlation coefficients were calculated using paired subsets of patients with available measurements for both the relevant TET parameter and the corresponding coagulation parameter. The analysis was exploratory. Abbreviations: LT, T lymphocytes; LB, B lymphocytes; NK, natural killer cells; G, granulocytes; M, monocytes; L, lymphocytes; TET, ten-eleven translocation protein.

Multiple moderate positive Spearman rank correlations (R = 0.25–0.49) were observed between TET parameters and aPTT and INR (Table 4); nominally significant correlations were at least as frequent for INR as for aPTT, with the strongest associations seen for monocyte-based TET indices and for NK-cell TET3. No significant associations were observed between TET parameters and the classical composite inflammatory indices (e.g., NLR, SII, SIRI), suggesting that the TET signal is not a simple reflection of leukocyte-count-derived inflammatory burden. These correlations were performed in the subset of patients with paired TET and coagulation measurements and are reported as exploratory; they should not be regarded as predictive. Accordingly, the continuous analysis did not confirm an aPTT-preferential pattern; the aPTT-specific signal was restricted to the categorical (median-split) analysis (Table 3). Figure 1 summarizes the Spearman correlations between TET parameters and the plasma coagulation parameters aPTT and INR in a heatmap format, facilitating rapid visual identification of the main association patterns across leukocyte subsets and TET isoforms.

### 3.5. Coagulation and TET Parameters According to Atrial Fibrillation

Patients were compared according to documented AF/AFL (electrocardiographic or device evidence, or a recorded history): 48 had AF/AFL (46 atrial fibrillation, two atrial flutter) and 59 did not. By rhythm at the time of sampling, 33 of the 107 patients were in atrial fibrillation, and 74 were in sinus rhythm; of the 48 with a documented AF/AFL history, 33 were in AF at sampling, and 15 were in sinus rhythm at sampling. AF/AFL was strongly associated with DOAC exposure (37/48 [77%] vs. 2/59 [3.4%]), so the two classifications are not independent. Compared with patients without AF/AFL, those with AF/AFL were older and had longer aPTT, higher INR, hs-CRP and CRP-to-neutrophil ratio, and lower platelet count and TAPSE. No statistically significant differences in TET1–3 values were found between the groups.

In forward stepwise multiple regression with the coagulation parameters as dependent variables, no TET parameter was associated with aPTT; some TET indices showed nominal associations with INR and with platelet count. Because many intercorrelated TET predictors were entered relative to the number of complete cases, the models are overfit, and none was corrected for multiplicity; these associations are exploratory and hypothesis-generating.

### 3.6. Clinical Phase as an Exploratory Modifier

In exploratory analyses stratified by clinical phase, correlations between TET parameters and plasma coagulation parameters were more numerous and stronger in clinically stable HFrEF than during worsening or decompensated HFrEF, in which only isolated associations were observed. Given the reduced subgroup sizes and the exploratory nature of these comparisons, this pattern is presented for descriptive purposes only.

These exploratory findings suggest that clinical phase may influence the detectability of immune–hemostatic associations.

## 4. Discussion

### 4.1. Principal Findings

In this two-center cross-sectional study of patients with HFrEF, we made four principal observations. First, TET protein expression was higher in selected leukocyte subpopulations among patients receiving DOACs. Second, higher aPTT was associated with higher TET expression in T cells, B cells, NK cells, and selected leukocyte indices. Third, the aPTT-specific signal was evident in the median-stratified (above- vs. below-median) analyses, whereas in the continuous correlation analysis TET expression was associated with both aPTT and INR. Fourth, associations appeared stronger in clinically stable HFrEF than during worsening or decompensated HFrEF.

### 4.2. Novelty and Clinical Relevance

To our knowledge, this is among the first clinical studies to link the protein expression of all three TET isoforms in multiple peripheral leukocyte subpopulations with plasma coagulation parameters in HFrEF. Three features support its translational relevance. The signal spans both adaptive (T and B lymphocytes) and innate (NK cells, monocytes) immune lineages, arguing against a single-lineage artifact. The TET parameters did not simply mirror classical composite inflammatory indices (NLR, SII, SIRI), indicating that TET-related immune-cell phenotyping may carry non-redundant biological information beyond leukocyte-count-derived markers. Finally, the integration of cellular immune phenotyping with clinically available hemostatic tests, in DOAC-treated patients sampled under steady-state trough conditions, makes the observed associations interpretable and clinically testable. These observations support a testable immune–hemostatic phenotyping hypothesis in HFrEF; they do not indicate that TET measurement is ready for clinical decision-making.

These findings should be interpreted within the boundary of what was measured. The study assessed TET protein expression only; it did not assess TET enzymatic activity, 5-methylcytosine or 5-hydroxymethylcytosine, global or locus-specific DNA methylation, or clonal hematopoiesis. The results should therefore be read as cell-subset-specific TET protein expression acting as an immune-cell phenotypic marker, not as evidence of epigenetic regulation of coagulation. Twelve-month rehospitalization and all-cause mortality were ascertained for all 107 participants and are reported as cohort characteristics (Table 1: rehospitalization 43.6% vs. 18.9% and all-cause mortality 28.2% vs. 17.6% in patients with vs. without DOAC exposure); however, the study was not powered to relate TET indices to these events and no time-to-event analysis of TET expression was performed. Functional validation of downstream TET activity and an adequately powered longitudinal TET–outcome analysis will therefore be required before clinical significance can be established.

The three isoforms are not biologically interchangeable. TET2 is the isoform most firmly linked to cardiovascular disease, principally through clonal hematopoiesis of indeterminate potential and inflammasome-dependent inflammation [26,27,28,29,30], whereas TET1 and TET3 have been implicated chiefly in lymphocyte developmental and fate decisions [21,23,25]. Current evidence does not, however, establish a direct relationship between TET1 or TET3 and aPTT or INR. The apparent predominance of TET1- and TET3-related signals in our data should be interpreted cautiously: it may reflect cell-subset-specific expression, differential transcriptional regulation, cell-activation state, subpopulation composition, clinical confounding, or multiple testing, and we did not formally test for differences between isoforms. It should therefore be regarded as an exploratory observation rather than evidence of a stronger biological effect of any single isoform.

### 4.3. Interpretation of the aPTT Signal

Biologically, aPTT is a clinically available, integrative test sensitive to the intrinsic and contact-activation pathways, and the contact and kallikrein–kinin systems form a molecular bridge between coagulation, fibrinolysis, and inflammation [40]. Activation of the contact and complement systems has been described in acute and chronic heart failure [41,42], and TET2-related mechanisms have been linked to venous thromboembolism [30], lending plausibility to a link between an immune-cell TET phenotype and a contact-pathway-sensitive coagulation phenotype. Because this aPTT-specific pattern was most apparent in the median-stratified analysis (Table 3) rather than in the continuous correlations—in which both aPTT and INR were associated with TET expression—we do not infer a contribution of hepatic synthetic function from these data. Contact-pathway markers, complement-activation markers, thrombin generation, individual coagulation factors, and anti-Xa concentrations were not measured; these interpretations therefore remain hypotheses to be tested rather than conclusions supported by the present data.

### 4.4. Relation to DOAC Exposure

The interpretation of differences in the DOAC group requires particular caution. DOAC exposure was clinically indicated and strongly associated with AF/AFL and with an older clinical phenotype, so it identified a distinct clinical and biological subgroup rather than a randomized intervention. The observed TET differences cannot be attributed directly to DOAC therapy, and the data should not be read as evidence that DOACs regulate TET expression. Consistent with this, exploratory multivariable models adjusting for age, AF, platelet count, hs-CRP, and composite inflammatory indices did not reach significance; with only 39 DOAC-treated patients, these models were underpowered and neither confirm nor exclude an independent association. These findings justify prospective studies incorporating anti-Xa activity, DOAC plasma concentrations, thrombin generation, and serial immune profiling.

### 4.5. Clinical Phase of HFrEF

The stronger associations in stable HFrEF are consistent with the clinical dynamics of decompensation. Acute decompensation may introduce substantial biological noise through neurohormonal activation, acute-phase inflammation, congestion, renal dysfunction, and treatment intensification, all of which may obscure more stable regulatory features of immune-cell biology; preanalytical heterogeneity related to sampling timing during decompensation may contribute further. This interpretation is exploratory and motivates serial sampling in strictly defined clinical states in future work [2,6].

### 4.6. Strengths

The study has several strengths: a two-center clinical cohort; a well-defined, guideline-managed HFrEF population; simultaneous assessment of all three TET isoforms across multiple leukocyte subpopulations; a reproducible flow-cytometric protocol with daily calibration, standardized cell input, titrated antibodies, and a GMFI ratio that normalizes for instrument drift and background [39], applied without modification from the validated protocol used in our prior HFrEF study [36], which supports methodological continuity and cross-cohort reproducibility of the TET indices; blood sampling in DOAC-treated patients under steady-state trough conditions, which limits pharmacodynamic distortion of coagulation tests [34,35]; and the integration of cellular immune markers with plasma coagulation parameters, with clinically relevant stratification by DOAC exposure and disease phase, in a single dataset.

### 4.7. Limitations

Several limitations should be acknowledged concisely. The design is cross-sectional and observational, precluding causal inference; the sample size was moderate; and the DOAC and non-DOAC groups differed substantially at baseline, with a marked AF/AFL imbalance, so confounding by indication cannot be excluded and power for multivariable adjustment was limited. The analyses spanned numerous subpopulations, three isoforms, and several endpoints without correction for multiple comparisons, so the correlation and subgroup analyses are exploratory; the aPTT median threshold is statistically convenient and not clinically established. DOAC plasma concentrations and anti-Xa/anti-IIa activity, thrombin generation, and contact-pathway and complement-activation markers were not measured, and we did not assess 5-hydroxymethylcytosine, DNA methylation, TET enzymatic activity, or clonal hematopoiesis; the findings therefore reflect antibody-accessible TET epitope abundance rather than enzymatic activity, and the polyclonal antibodies were not validated in TET-knockout systems. There was no external validation cohort, and blood was sampled at a single time point. In addition, hemostatic status was characterized only by aPTT and INR, so a comprehensive hemostatic profile was not obtained; the multivariable (forward stepwise) analyses are exploratory and were not corrected for multiple comparisons, and given the moderate sample, incomplete TET data and the intercorrelation among TET indices the retained determinants require confirmation in larger, prospectively phenotyped cohorts; and the comparator group defined as not receiving a DOAC included seven patients receiving a vitamin K antagonist, which may influence INR, so a sensitivity analysis excluding these patients is advisable.

Looking forward, prospective longitudinal validation is needed, ideally with serial sampling during decompensation and after stabilization, integration with thrombin generation and contact-pathway and complement-activation markers, and direct assessment of TET-axis products together with CHIP/TET2 analysis. Adequately powered models linking TET-related immune-cell phenotyping to bleeding, thromboembolism, heart failure hospitalization, and mortality will be required before any future biomarker-driven phenotyping can be considered.

## 5. Conclusions

In this two-center, cross-sectional study of patients with HFrEF, peripheral leukocyte TET1–3 protein expression was associated with routinely available plasma coagulation parameters (aPTT and INR), and these associations were not mirrored by composite leukocyte-derived inflammatory indices. Because the design is cross-sectional and exploratory, the sample is moderate with incomplete TET data, and DOAC exposure is confounded with atrial fibrillation, these findings are hypothesis-generating and do not establish a causal or mechanistic link. Prospective, adequately powered studies are warranted to test whether TET-related immune-cell phenotyping carries clinically useful immune–coagulation information.

## Figures and Tables

**Table 1:** Baseline clinical characteristics of patients with HFrEF according to DOAC exposure. Values are mean ± SD or *n* (%). Bold *p*-values are nominally significant (*p* < 0.05). Between-group comparisons used the Student *t*-test, Mann–Whitney U test, or chi-square/Fisher exact test as appropriate; analyses are exploratory, and *p*-values are nominal (uncorrected for multiple comparisons).

Parameter	With DOAC (*n* = 39; 36.4%)	Without DOAC (*n* = 68; 63.6%)	*p*
**Age (years)**	**70.6 ± 11.03**	**64.29 ± 13.16**	**0.013**
CHF exacerbation, *n* (%)	14 (35.9)	23 (33.8)	0.828
**Atrial fibrillation (AF), *n* (%)**	**26 (66.7)**	**7 (10.3)**	**<0.001**
**Rehospitalization within 12 months, *n* (%)**	**17 (43.6)**	**13 (18.9)**	**0.003**
One-year mortality, *n* (%)	11 (28.2)	12 (17.6)	0.123
N-terminal pro-B-type natriuretic peptide (NT-proBNP) [pg/mL]	4339.4 ± 6601.7	3485.09 ± 5365.6	0.468
Left ventricular end–diastolic diameter (LVEDD) [mm]	60.4 ± 9.31	61.68 ± 7.55	0.446
Left ventricular ejection fraction (LVEF) [%]	28.9 ± 7.56	29.25 ± 7.48	0.803
**Tricuspid annular plane systolic excursion (TAPSE)**	**16.3 ± 4.56**	**18.10 ± 4.62**	**0.048**
**Activated partial thromboplastin time (aPTT) [s]**	**36.2 ± 10.2**	**30.78 ± 7.36**	**0.002**
International normalized ratio (INR)	1.3 ± 0.46	1.13 ± 0.38	0.062
White blood cell count (WBC) [×10^9^/L]	7.5 ± 2.16	8.30 ± 2.90	0.125
Hemoglobin (HGB) [g/dL]	14.3 ± 2.22	14.12 ± 1.73	0.628
**Platelet count (PLT) [×10^9^/L]**	**194.8 ± 71.35**	**230.15 ± 69.53**	**0.014**
**Absolute neutrophil count (NEU) [×10^9^/L]**	**5.3 ± 2.38**	**6.68 ± 3.09**	**0.022**
Absolute lymphocyte count (LYM) [×10^9^/L]	2.3 ± 1.28	2.18 ± 1.20	0.595
Absolute monocyte count (MONO) [×10^9^/L]	0.8 ± 0.42	0.83 ± 0.41	0.608
Sodium (Na+) [mmol/L]	140.6 ± 2.97	140.29 ± 3.17	0.607
Potassium (K+) [mmol/L]	4.4 ± 0.46	4.41 ± 0.45	0.582
Creatinine [mg/dL]	1.1 ± 0.38	1.09 ± 0.28	0.995
Estimated glomerular filtration rate by CKD-EPI (eGFR)	71.8 ± 21.06	72.03 ± 22.42	0.9530
Glucose [mg/dL]	115.2 ± 30.9	112.43 ± 27.4	0.634
Aspartate aminotransferase (AST)	30.5 ± 18.12	26.90 ± 12.78	0.228
Alanine aminotransferase (ALT)	30.3 ± 20.07	28.79 ± 18.56	0.699
**High-sensitivity C-reactive protein (hs-CRP) [mg/L]**	**12.1 ± 19.14**	**6.46 ± 8.92**	**0.040**
CRP/LYM	5.7 ± 7.78	4.43 ± 9.25	0.462
**CRP/NEU**	**2.3 ± 3.70**	**0.96 ± 0.96**	**0.008**
**Neutrophil-to-lymphocyte ratio (NLR)**	**2.8 ± 1.52**	**3.69 ± 2.37**	**0.031**
Neutrophil-to-platelet ratio (NPR)	0.0 ± 0.02	0.03 ± 0.02	0.999
Lymphocyte-to-monocyte ratio (LMR)	3.2 ± 1.38	2.96 ± 1.64	0.510
PLT/HGB	14.3 ± 7.82	16.50 ± 5.33	0.084
**Systemic inflammation response index (SIRI)**	**2.1 ± 1.4**	**3.31 ± 3.35**	**0.033**
**Systemic immune-inflammation index (SII)**	**527.9 ± 373.55**	**895.13 ± 850.23**	**0.012**
IBI	33.0 ± 48.73	38.35 ± 119.13	0.791

**Table 2:** TET protein expression by leukocyte subpopulation according to DOAC exposure, grouped by cell type. Values are mean ± SD of the GMFI ratio (test/control); bold *p*-values are nominally significant. Mann–Whitney U test; analyses are exploratory (nominal, uncorrected *p*-values). Abbreviations: G, granulocytes; L, lymphocytes; LB, B lymphocytes; LT, T lymphocytes; M, monocytes; NK, natural killer cells; PW, percentage-weighted; TET, ten-eleven translocation.

Parameter	With DOAC (*n* = 39; 36.4%)	Without DOAC (*n* = 68; 63.6%)	*p*
**LT_TET1**	**1.7 ± 1.12**	**1.17 ± 0.29**	**0.005**
LT_TET2	2.2 ± 1.36	1.83 ± 0.87	0.186
**LT_TET3**	**2.3 ± 1.77**	**1.43 ± 0.69**	**0.024**
**LB_TET1**	**1.5 ± 0.91**	**1.11 ± 0.27**	**0.005**
LB_TET2	2.9 ± 2.47	2.11 ± 1.25	0.098
**LB_TET3**	**2.1 ± 1.75**	**1.30 ± 0.62**	**0.011**
**NK_TET1**	**2.0 ± 1.63**	**1.24 ± 0.68**	**0.022**
**NK_TET2**	**2.6 ± 2.25**	**1.74 ± 1.05**	**0.036**
**NK_TET3**	**3.3 ± 3.19**	**1.35 ± 1.11**	**0.007**
**PW_LT_TET1**	**0.3 ± 0.28**	**0.18 ± 0.10**	**0.049**
PW_LT_TET2	0.4 ± 0.32	0.26 ± 0.24	0.154
**PW_LT_TET3**	**0.4 ± 0.39**	**0.23 ± 0.18**	**0.044**
**PW_LB_TET1**	**0.1 ± 0.13**	**0.05 ± 0.04**	**0.007**
PW_LB_TET2	0.2 ± 0.29	0.09 ± 0.13	0.051
**PW_LB_TET3**	**0.2 ± 0.23**	**0.06 ± 0.07**	**0.010**
PW_NK_TET1	0.0 ± 0.02	0.01 ± 0.02	0.223
**PW_NK_TET2**	**0.0 ± 0.03**	**0.02 ± 0.02**	**0.049**
**PW_NK_TET3**	**0.0 ± 0.04**	**0.02 ± 0.02**	**0.010**
**G_TET1 index**	**1.3 ± 0.54**	**1.14 ± 0.18**	**0.011**
G_TET2 index	1.2 ± 0.53	1.36 ± 1.31	0.444
G_TET3 index	1.9 ± 1.35	1.61 ± 1.97	0.435
**M_TET1 index**	**1.9 ± 1.21**	**1.33 ± 0.41**	**0.002**
M_TET2 index	1.9 ± 1.39	1.56 ± 0.80	0.168
**M_TET3 index**	**2.8 ± 2.22**	**1.59 ± 1.21**	**0.002**
L_TET2 index	2.0 ± 1.25	1.89 ± 1.18	0.764
**L_TET3 index**	**2.2 ± 1.51**	**1.43 ± 0.83**	**0.003**

**Table 3:** TET protein expression according to aPTT stratified at the cohort median (30.7 s). Values are mean ± SD; bold *p*-values are nominally significant (Mann–Whitney U test; exploratory, nominal *p*-values). The median threshold is statistically convenient and has no established clinical cut-off. Abbreviations as in Table 2.

Parameter	aPTT ≥ 30.7 s (*n* = 53)	aPTT < 30.7 s (*n* = 54)	*p*
**LT_TET1**	**1.57 ± 1.00**	**1.15 ± 0.18**	**0.027**
**LT_TET2**	**2.26 ± 1.40**	**1.66 ± 0.49**	**0.017**
**LT_TET3**	**2.09 ± 1.62**	**1.37 ± 0.48**	**0.047**
**LB_TET1**	**1.40 ± 0.82**	**1.12 ± 0.24**	**0.048**
**LB_TET2**	**2.93 ± 2.36**	**1.86 ± 0.73**	**0.010**
**LB_TET3**	**1.96 ± 1.60**	**1.24 ± 0.37**	**0.020**
**NK_TET1**	**1.83 ± 1.55**	**1.17 ± 0.25**	**0.031**
**NK_TET2**	**2.60 ± 2.13**	**1.53 ± 0.59**	**0.004**
**NK_TET3**	**2.96 ± 3.74**	**1.17 ± 0.34**	**0.010**
PW_LT_TET1	0.24 ± 0.25	0.19 ± 0.08	0.286
PW_LT_TET2	0.35 ± 0.36	0.23 ± 0.13	0.063
PW_LT_TET3	0.35 ± 0.38	0.23 ± 0.11	0.138
PW_LB_TET1	0.08 ± 0.12	0.05 ± 0.04	0.110
**PW_LB_TET2**	**0.18 ± 0.28**	**0.08 ± 0.07**	**0.033**
**PW_LB_TET3**	**0.14 ± 0.21**	**0.05 ± 0.05**	**0.035**
PW_NK_TET1	0.02 ± 0.02	0.02 ± 0.02	0.713
PW_NK_TET2	0.03 ± 0.03	0.03 ± 0.02	0.686
PW_NK_TET3	0.03 ± 0.04	0.02 ± 0.02	0.213
G_TET1 index	1.26 ± 0.46	1.13 ± 0.20	0.097
G_TET2 index	1.35 ± 1.20	1.28 ± 1.16	0.772
G_TET3 index	2.11 ± 2.53	1.37 ± 0.52	0.088
**M_TET1 index**	**1.68 ± 1.10**	**1.33 ± 0.30**	**0.042**
**M_TET2 index**	**1.98 ± 1.35**	**1.40 ± 0.52**	**0.011**
M_TET3 index	2.26 ± 2.02	1.76 ± 1.36	0.212
**L_TET2 index**	**2.21 ± 1.54**	**1.67 ± 0.75**	**0.042**
L_TET3 index	1.93 ± 1.39	1.52 ± 0.90	0.144

**Table 4:** Spearman rank correlations between TET parameters and plasma coagulation parameters, showing the correlation of each TET parameter with aPTT and with INR in patients with paired TET and coagulation measurements. Bold *p*-values are nominally significant (*p* < 0.05); analyses are exploratory and uncorrected for multiple comparisons. Abbreviations: aPTT, activated partial thromboplastin time; INR, international normalized ratio; others as in Table 2.

Correlation	Spearman’s R	*p*
LT_TET1 & aPTT	0.25	0.071
**LT_TET1 & INR**	**0.37**	**0.005**
LT_TET2 & aPTT	0.13	0.297
LT_TET2 & INR	0.07	0.588
LT_TET3 & aPTT	0.29	0.061
**LT_TET3 & INR**	**0.31**	**0.043**
LB_TET1 & aPTT	0.10	0.432
LB_TET1 & INR	0.18	0.154
LB_TET2 & aPTT	0.25	0.052
LB_TET2 & INR	0.21	0.099
LB_TET3 & aPTT	0.21	0.148
LB_TET3 & INR	0.14	0.314
NK_TET1 & aPTT	0.09	0.504
NK_TET1 & INR	0.23	0.099
NK_TET2 & aPTT	0.23	0.068
NK_TET2 & INR	0.20	0.115
**NK_TET3 & aPTT**	**0.40**	**0.003**
**NK_TET3 & INR**	**0.33**	**0.019**
G_TET1 index & aPTT	0.24	0.087
G_TET1 index & INR	0.19	0.175
G_TET2 index & aPTT	0.27	0.053
G_TET2 index & INR	-0.03	0.842
G_TET3 index & aPTT	0.27	0.085
**G_TET3 index & INR**	**0.31**	**0.045**
**M_TET1 index & aPTT**	**0.39**	**0.005**
**M_TET1 index & INR**	**0.49**	**0.001**
**M_TET2 index & aPTT**	**0.45**	**0.001**
M_TET2 index & INR	0.23	0.103
M_TET3 index & aPTT	0.30	0.061
**M_TET3 index & INR**	**0.41**	**0.008**
**L_TET1 index & aPTT**	**0.30**	**0.035**
**L_TET1 index & INR**	**0.38**	**0.006**
**L_TET2 index & aPTT**	**0.39**	**0.005**
L_TET2 index & INR	0.23	0.101
L_TET3 index & aPTT	0.19	0.242
**L_TET3 index & INR**	**0.40**	**0.010**

## Data Availability

The de-identified dataset supporting the findings is not publicly available owing to institutional and legal restrictions on sharing individual-level clinical data and is available from the corresponding author on reasonable request.
